# Supplementary material for: Eustress with H2O2 Facilitates Plant Growth by Improving Tolerance to Salt Stress in Two Wheat Cultivars
Source: Plants (Basel). 2019 Aug 27;8(9):303. doi: 10.3390/plants8090303 (PMC6783893; doi:10.3390/plants8090303)
Supplement: Supplementary file 1 [file plants-08-00303-s001.pdf]

Table S1. Inter-simple sequence repeat (ISSR) analysis of polymorphic bands for eight treatments using six primers. T1 = W + W (0%); T2 = H_2O_2 + W (0%); T3 = W + SW (35%); T4 = H_2O_2 + SW (35%).

			Gemmiza 11				Misr 1			
**Primer name**	**P%**	**Base pair (bp)**	**T_1**	**T_2**	**T_3**	**T_4**	**T_1**	**T_2**	**T_3**	**T_4**
ISSR-1	72.22%	1150	(+)	(+)	(-)	(+)	(+)	(+)	(-)	(+)
		820	(+)	(-)	(-)	(-)	(-)	(-)	(+)*	(-)
		740	(-)	(-)	(-)	(-)	(-)	(-)	(-)	(+)*
		480	(+)	(-)	(-)	(-)	(-)	(+)*	(+)*	(+)*
		430	(-)	(-)	(-)	(+)*	(-)	(-)	(-)	(+)*
		420	(-)	(-)	(-)	(+)	(-)	(+)*	(-)	(+)*
		390	(-)	(-)	(-)	(+)	(+)	(+)	(-)	(+)
		320	(-)	(+)*	(-)	(+)*	(-)	(+)*	(-)	(+)*
		280	(-)	(-)	(+)*	(-)	(-)	(-)	(+)*	(-)
		230	(-)	(+)*	(-)	(+)*	(-)	(+)*	(-)	(+)*
		180	(+)	(-)	(+)	(+)	(-)	(+)*	(+)*	(+)*
		160	(-)	(-)	(-)	(-)	(-)	(+)*	(-)	(+)*
		140	(-)	(-)	(-)	(-)	(-)	(-)	(+)*	(-)
**New bands (*)**			**0**	**2**	**1**	**3**	**0**	**6**	**5**	**8**
**Total bands**			**4**	**3**	**2**	**7**	**2**	**8**	**5**	**10**
			**Gemmiza 11**				**Misr 1**			
**Primer name**	**P%**	**Base pair (bp)**	**T_1**	**T_2**	**T_3**	**T_4**	**T_1**	**T_2**	**T_3**	**T_4**
ISSR-2	54.54%	1200	(-)	(-)	(-)	(+)*	(-)	(-)	(-)	(+)*
		480	(-)	(-)	(-)	(-)	(-)	(-)	(+)*	(-)
		350	(-)	(-)	(+)*	(-)	(-)	(-)	(+)*	(-)
		330	(+)	(+)	(-)	(+)	(+)	(+)	(-)	(+)
		300	(-)	(-)	(-)	(-)	(-)	(+)*	(+)*	(+)*
		230	(-)	(+)*	(-)	(-)	(-)	(+)*	(-)	(+)*
**New bands (*)**			**0**	**1**	**1**	**1**	**0**	**2**	**3**	**3**
**Total bands**			**1**	**2**	**1**	**2**	**1**	**3**	**3**	**4**
			**Gemmiza 11**				**Misr 1**			
**Primer name**	**P%**	**Base pair (bp)**	**T_1**	**T_2**	**T_3**	**T_4**	**T_1**	**T_2**	**T_3**	**T_4**
ISSR-3	92%	2000	(+)	(+)	(-)	(-)	(+)	(+)	(-)	(+)
		1700	(-)	(+)*	(+)*	(+)*	(-)	(+)*	(+)*	(+)*
		1550	(-)	(+)	(-)	(+)	(-)	(-)	(-)	(+)
		1450	(+)	(+)	(-)	(+)	(+)	(+)	(-)	(+)
		1300	(-)	(+)*	(+)*	(+)*	(-)	(+)*	(+)*	(+)*
		1200	(-)	(+)	(+)	(+)	(-)	(+)*	(-)	(+)*
		1150	(-)	(-)	(-)	(-)	(-)	(+)*	(-)	(+)*
		1050	(+)	(+)	(-)	(+)	(-)	(-)	(-)	(+)*
		970	(-)	(+)	(+)*	(+)*	(+)	(+)	(-)	(+)
		870	(+)	(+)	(-)	(+)	(-)	(+)	(+)	(+)
		840	(-)	(-)	(-)	(+)	(-)	(+)*	(-)	(+)*
		820	(-)	(+)	(+)	(-)	(-)	(-)	(+)*	(-)
		750	(+)	(-)	(-)	(-)	(-)	(-)	(-)	(+)*
		720	(+)	(-)	(-)	(+)	(+)	(-)	(+)	(-)
		570	(-)	(+)	(+)	(-)	(-)	(+)*	(-)	(+)*
		520	(-)	(+)	(+)	(+)	(+)	(+)	(-)	(+)
		480	(-)	(+)	(+)	(+)	(+)	(-)	(+)	(+)
		440	(+)	(-)	(+)	(+)	(+)	(-)	(-)	(-)
		380	(+)	(+)	(-)	(+)	(+)	(+)	(-)	(+)
		320	(-)	(+)*	(-)	(+)*	(-)	(+)*	(-)	(+)*
		270	(+)	(+)	(+)	(+)	(-)	(-)	(+)*	(-)
		230	(+)	(+)	(-)	(+)	(+)	(+)	(-)	(+)
		210	(+)	(-)	(-)	(+)	(-)	(-)	(-)	(+)*

**New bands (*)**			**0**	**3**	**3**	**4**	**0**	**7**	**4**	**10**
**Total bands**			**11**	**17**	**10**	**18**	**9**	**14**	**7**	**19**

			**Gemmiza 11**				**Misr 1**			
**Primer name**	**P%**	**Base pair (bp)**	**T_1**	**T_2**	**T_3**	**T_4**	**T_1**	**T_2**	**T_3**	**T_4**
**ISSR-4**	**82.35%**	**1450**	(-)	(+)	(+)	(+)	(-)	(-)	(+)*	(-)
		**1100**	(-)	(+)*	(-)	(+)*	(-)	(+)*	(-)	(+)*
		**1000**	(-)	(+)*	(-)	(+)*	(-)	(+)*	(+)*	(+)*
		**900**	(+)	(+)	(-)	(+)	(+)	(+)	(-)	(+)
		**800**	(-)	(+)	(+)	(+)	(+)	(+)	(+)	(+)
		**740**	(-)	(-)	(+)*	(-)	(-)	(+)*	(-)	(+)*
		**680**	(-)	(-)	(+)	(+)	(-)	(-)	(-)	(-)
		**620**	(-)	(-)	(+)	(+)	(+)	(+)	(-)	(+)
		**550**	(-)	(-)	(+)*	(-)	(-)	(+)*	(+)*	(+)*
		**510**	(+)	(-)	(-)	(-)	(-)	(+)*	(-)	(+)*
		**430**	(+)	(-)	(-)	(+)	(-)	(-)	(-)	(+)*
		**400**	(-)	(+)	(+)	(+)	(-)	(-)	(-)	(+)*
		**360**	(-)	(-)	(-)	(+)	(+)	(+)	(-)	(+)
		**260**	(-)	(+)	(-)	(+)	(-)	(+)*	(+)*	(+)*
**New bands (*)**			**0**	**2**	**2**	**2**	**0**	**6**	**4**	**8**
**Total bands**			**3**	**7**	**7**	**11**	**4**	**10**	**5**	**12**

			**Gemmiza 11**				**Misr 1**			
**Primer name**	**P%**	**Base pair (bp)**	**T_1**	**T_2**	**T_3**	**T_4**	**T_1**	**T_2**	**T_3**	**T_4**
**ISSR-5**	**84.21%**	**1350**	(+)	(-)	(-)	(-)	(-)	(-)	(+)*	(-)
		**1100**	(-)	(+)*	(-)	(+)*	(-)	(+)*	(-)	(+)*
		**1000**	(+)	(+)	(-)	(-)	(+)	(+)	(-)	(+)
		**910**	(-)	(-)	(-)	(-)	(-)	(+)*	(-)	(+)*
		**850**	(+)	(-)	(+)	(-)	(+)	(-)	(-)	(-)
		**800**	(-)	(+)	(-)	(-)	(+)	(+)	(-)	(+)
		**710**	(+)	(-)	(+)	(-)	(-)	(-)	(+)*	(-)
		**660**	(+)	(+)	(+)	(+)	(+)	(+)	(-)	(+)
		**620**	(-)	(-)	(+)*	(-)	(-)	(-)	(+)*	(-)
		**540**	(-)	(-)	(+)*	(-)	(-)	(-)	(+)*	(-)
		**390**	(-)	(+)*	(-)	(+)*	(-)	(+)*	(-)	(+)*
		**360**	(-)	(-)	(-)	(-)	(-)	(+)*	(-)	(+)*
		**340**	(-)	(-)	(-)	(+)	(-)	(-)	(-)	(+)*
		**320**	(-)	(+)	(-)	(+)	(-)	(+)*	(-)	(+)*
		**250**	(-)	(-)	(-)	(-)	(+)	(-)	(-)	(+)
		**230**	(-)	(+)	(-)	(+)	(-)	(+)*	(-)	(+)*
**New bands (*)**			**0**	**2**	**2**	**2**	**0**	**6**	**4**	**7**
**Total bands**			**5**	**7**	**5**	**6**	**5**	**9**	**4**	**11**

			**Gemmiza 11**				**Misr 1**			
**Primer name**	**P%**	**Base pair (bp)**	**T_1**	**T_2**	**T_3**	**T_4**	**T_1**	**T_2**	**T_3**	**T_4**
**ISSR-6**	**81.81%**	**970**	(+)	(-)	(+)	(+)	(+)	(+)	(-)	(+)
		**760**	(-)	(+)	(-)	(-)	(-)	(-)	(+)*	(-)
		**590**	(+)	(+)	(-)	(+)	(-)	(-)	(-)	(+)*
		**440**	(+)	(+)	(-)	(-)	(+)	(+)	(+)	(+)
		**380**	(+)	(+)	(+)	(+)	(-)	(-)	(+)*	(-)
		**320**	(-)	(+)*	(-)	(+)*	(-)	(+)*	(-)	(+)*
		**260**	(+)	(-)	(+)	(+)	(+)	(+)	(-)	(+)
		**230**	(-)	(+)	(-)	(-)	(-)	(-)	(-)	(+)*
		**160**	(-)	(+)*	(-)	(+)*	(-)	(+)*	(-)	(+)*
**New bands(*)**			**0**	**2**	**0**	**2**	**0**	**2**	**2**	**4**
**Total bands**			**5**	**7**	**3**	**6**	**3**	**5**	**3**	**7**
